# Laboratory tests as short-term correlates of stroke

**DOI:** 10.1186/s12883-016-0619-y

**Published:** 2016-07-21

**Authors:** Trevor Sughrue, Michael A. Swiernik, Yang Huang, James P. Brody

**Affiliations:** Kaiser Permanente Southern California, San Diego, CA USA; Department of Biomedical Engineering, University of California—Irvine, Irvine, CA 92697-2715 USA; Department of Biomedical Engineering, Henry Samueli School of Engineering, University of California, Irvine, CA 92603 USA

## Abstract

**Background:**

The widespread adoption of electronic health records provides new opportunities to better predict which patients are likely to suffer a stroke. Using electronic health records, we assessed the correlation of different laboratory tests to future occurrences of a stroke.

**Methods:**

We examined the electronic health records of 2.4 million people over a two year time span. These records contained 26,964 diagnoses of stroke. Using Cox regression analysis, we measured whether any one of 1796 different laboratory tests were effectively correlated with a future diagnosis of stroke.

**Results:**

We identified 38 different laboratory tests that had significant short-term (two year) prognostic value for a future diagnosis of stroke. For each of the 38 laboratory tests we also compiled the Kaplan-Meier survival curve, and relative risk ratio that the test confers.

**Conclusion:**

Several dozen laboratory tests are effective short-term correlates of stroke.

**Electronic supplementary material:**

The online version of this article (doi:10.1186/s12883-016-0619-y) contains supplementary material, which is available to authorized users.

## Background

Several stroke risk scores have been developed to identify those with the highest risk of stroke [1–4]. These stroke risk scores are mostly based on information one could collect when taking a patient’s medical history. Identification of those most at risk for developing stroke would allow focused education on both reducing risk factors and recognizing signs of a stroke. Early recognition and treatment of stroke can substantially reduce both the direct and indirect costs of a stroke [5].

Electronic health records (EHRs) are rapidly being adopted by medical providers, and are now used by the majority of office-based physicians and hospitals in the US [6, 7]. These EHRs do not always provide easily computable information regarding a patient’s medical history, but do an excellent job of providing discrete data from laboratory tests, imaging studies, and pharmacy records. Thus an opportunity exists to develop a stroke risk score that one could compute from discrete data contained in EHRs.

The best known stroke risk score is the Framingham Stroke Risk Profile, developed as part of the Framingham Heart Study [1, 2]. This risk score was based on an analysis of 472 stroke events. The score computes the probability of developing a stroke within the next 10 years based on age, sex, systolic blood pressure, along with categorical factors disclosed by the patient when taking a medical history such as whether the patient smokes cigarettes or has been diagnosed with diabetes or atrial fibrillation.

Two other stroke risk scores have been published. First, a short term (3 year) measure of stroke risk was developed based on 188 strokes observed in data collected by the Cardiovascular Health Study [8, 9]. Second, a long term (two decades) stroke risk score was developed based on 282 strokes observed in a population of municipal employees in Israel as part of the Israel Ischemic Heart Disease Project [4].

Several different health outcomes have been predicted from electronic health records [10]. Examples include: the identification of drug-drug interactions [11, 12], computation of the genetic risk for diabetes [13], identification of diabetes medication that significantly increased risk of myocardial infarction [14] and prediction of the patients’ future risk of receiving a diagnosis of domestic abuse [15].

The purpose of this study is to identify laboratory tests that effectively correlate with the occurrence of stroke. This study is based on 26,954 observations of stroke in a one-year period from the electronic health records of a large managed care organization.

## Methods

We tested which laboratory tests were correlated with a future diagnosis of stroke using Cox Regression, controlling for age and sex differences. We used a database of electronic health records from Kaiser Permanente containing one year of patient records containing records for 2.4 million patients, 26,964 of whom received a diagnosis of stroke during the one year period. We compared laboratory test results that the 26,964 patients received several months before their diagnosis of stroke with the laboratory test results that the patients who never received a stroke diagnosis. From this comparison, we identified laboratory tests that had significantly different results in the two populations: those who would have a stroke within a few months and those who would not.

Kaiser Permanente (KP) is a non-profit health plan with 9.6 million members, and the largest region is Southern California with over 3.8 million members. KP offers comprehensive health care including outpatient and inpatient care, laboratory services, and pharmacies. Kaiser Permanente implemented electronic health records before 2009 [16, 17].

This is a retrospective data-only study. All data for this study were selected from members of Kaiser Permanente’s Southern California Health Plan who had continuous coverage over a recent one-year time span inclusive of 2013. Subjects were not systematically tested, but only received specific tests indicated by their medical condition. We limited the subject pool to only those patients that had received any form of care from Kaiser Permanente during 2013, including laboratory-only visits and both inpatient and outpatient encounters. Furthermore, we included only one laboratory result per type of test per month. We limited the data to one result per month to prevent over counting individuals who may be sampled more frequently, which would bias our results. Only discrete data elements of demographics, diagnoses, laboratory tests, and EKGs were extracted and analyzed, from which all Protected Health Information (PHIs) were removed during data extraction.

**Fig. 1:**
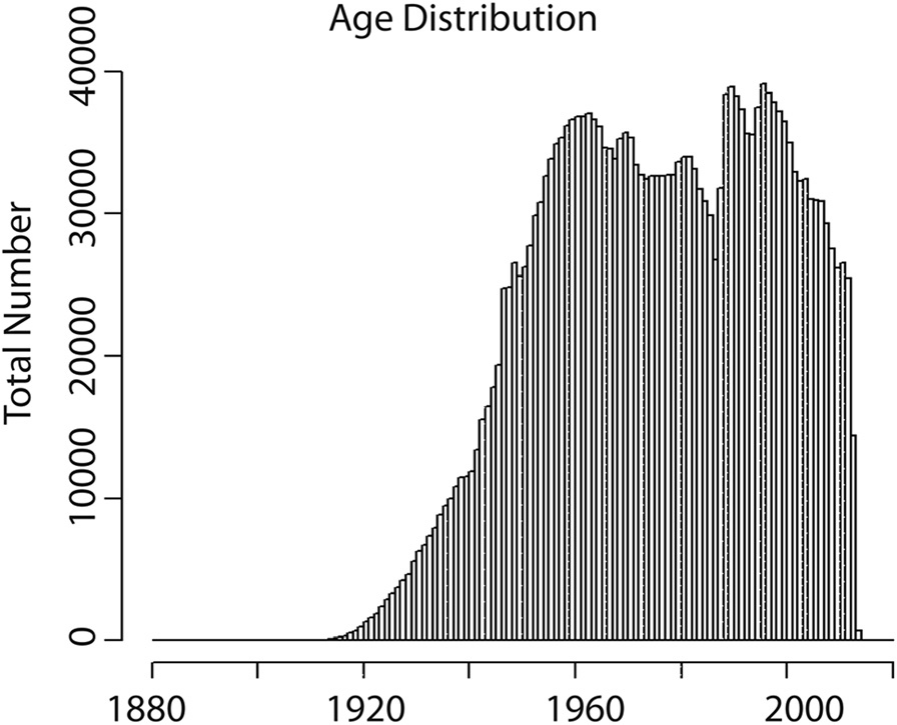
The age distribution of patients in the dataset

Overall, the study included 2,412,213 individuals. Of these 51 % (1,239,559) were female. The birth-year distribution of this population is shown in Fig. 1 and the frequency of self-reported race/ethnicity categories are shown in Additional file 1: Table S1.

### ICD-9 Codes

Diagnoses were identified by ICD-9 codes, and we included diagnoses that were coded for encounters or were added to problem lists during the time period. We included both ischemic and hemorrhagic strokes, and included all diagnoses with ICD-9 codes that began with 431, 433, and 434. The total number of strokes suffered by the cohort during this time period was 26,964. 95.17 % were ischemic strokes.

### LOINC codes

Laboratory tests and other procedures are classified using the Logical Observation and Identifiers Names and Codes (LOINC) system [18, 19]. This system standardizes electronic records of medical laboratory results. A total of 1796 LOINC codes were found in the Kaiser Permanente database. We tested whether each of these correlated with a future diagnosis of stroke. Of the 2,412,213 Southern California Kaiser Permanente members during the time period analyzed, 1,406,413 (58 %) had one or more LOINC record.

### Cox regression

We asked whether any of the 1796 laboratory tests had significant correlation with stroke. To determine whether any significant correlation exists, we used Cox proportional hazards model [20, 21]. The level of significance was conservatively set at 10^−2^ divided by the number of comparisons, 1796. We rounded this quotient down to 10^−6^, which was set as the level of significance. We supplied the patient’s age and sex as covariates to each of the 1796 regressions. Therefore, test results that are correlated with age alone will not be identified as significant correlates with stroke. We used the survival package in the R statistical software package [22, 23] to perform the regression. More advanced statistical approaches exist and could be applied to this problem [24].

We excluded some laboratory test results from our analysis. Some laboratory tests are repeated frequently on a patient. To prevent overweighting with these tests, we only included one of these measurements per month, using the median for a month of measurements as that month’s value. Cox Regression assumes independence between measurements, but we did not establish that measurements made one month apart are independent.

## Results

### Output table

Of the 1796 LOINC codes tested, 38 had a statistically significant correlation with a future diagnosis of stroke, indicated by a *p*-value less than 10^−6^. Tests with a positive correlation indicated that an increase in the lab value saw an increased chance of stroke. A negative number indicated that an increase in lab value correlated with a decreased chance of stroke. Laboratory tests that are significant correlates of stroke can be seen in Table 1. Since each laboratory test has a different mean and distribution of values, we calculated an adjusted coefficient that shows the change in the likelihood of stroke given a 1 % increase in lab values.

**Table 1:** List of laboratory tests that were statistically significant correlates of a later diagnosis of stroke

LOINC_ID	Adjusted coefficient	*P*-value	Name
2160-0	0.214	<1.0E-17	Creatinine [Mass/volume] in Serum or Plasma
2823-3	0.0725	<1.0E-17	Potassium [Moles/volume] in Serum or Plasma
4548-4	0.0163	<1.0E-17	Hemoglobin A1c/Hemoglobin.total in Blood
751-8	0.0129	<1.0E-17	Neutrophils [#/volume] in Blood by Automated count
3094-0	0.000801	<1.0E-17	Urea nitrogen [Mass/volume] in Serum or Plasma
770-8	0.000229	<1.0E-17	Neutrophils/100 leukocytes in Blood by Automated count
17865-7	0.0000519	<1.0E-17	Glucose [Mass/volume] in Serum or Plasma --8 h fasting
2345-7	0.0000315	<1.0E-17	Glucose [Mass/volume] in Serum or Plasma
27353-2	0.0000237	<1.0E-17	Glucose mean value [Mass/volume] in Blood Estimated from glycated hemoglobin
14957-5	0.00000201	<1.0E-17	Microalbumin [Mass/volume] in Urine
14959-1	0.00000132	<1.0E-17	Microalbumin/Creatinine [Mass Ratio] in Urine
33914-3	3.72E-15	<1.0E-17	Glomerular filtration rate/1.73 sq M.predicted by Creatinine-based formula (MDRD)
14135-8	0.000000994	2.22E-16	CD3 + CD8+ (T8 suppressor cells) cells [#/volume] in Blood
5902-2	0.00253	2.22E-16	Prothrombin time (PT) in Platelet poor plasma by Coagulation assay
42246-9	0.213	4.44E-16	Hemoglobin F/Hemoglobin.total in Blood by HPLC
742-7	0.359	8.44E-15	Monocytes [#/volume] in Blood by Automated count
711-2	2.99	8.44E-15	Eosinophils [#/volume] in Blood by Automated count
8636-3	0.0000123	6.89E-14	Q-T interval corrected
8633-0	0.0000704	1.17E-12	QRS duration
30934-4	0.00000147	3.10E-12	Natriuretic peptide B [Mass/volume] in Serum or Plasma
713-8	0.0163	7.54E-12	Eosinophils/100 leukocytes in Blood by Automated count
6768-6	0.0000145	1.38E-08	Alkaline phosphatase [Enzymatic activity/volume] in Serum or Plasma
8122-4	0.000000224	3.30E-08	CD3 cells [#/volume] in Blood
18518-1	0.0000775	5.97E-07	T wave axis.frontal plane Reference beat
2039-6	0.000871	0.00000895	Carcinoembryonic Ag [Mass/volume] in Serum or Plasma
1751-7	−0.105	<1.0E-17	Albumin [Mass/volume] in Serum or Plasma
2028-9	−0.00139	<1.0E-17	Carbon dioxide
4544-3	−0.00102	<1.0E-17	Hematocrit [Volume Fraction] of Blood by Automated count
736-9	−0.000697	<1.0E-17	Lymphocytes/100 leukocytes in Blood by Automated count
2085-9	−0.000303	<1.0E-17	Cholesterol in HDL [Mass/volume] in Serum or Plasma
2951-2	−0.000219	<1.0E-17	Sodium [Moles/volume] in Serum or Plasma
13457-7	−0.0000849	<1.0E-17	Cholesterol in LDL [Mass/volume] in Serum or Plasma by calculation
2089-1	−0.0000721	<1.0E-17	Cholesterol in LDL [Mass/volume] in Serum or Plasma
43396-1	−0.0000624	<1.0E-17	Cholesterol non HDL [Mass/volume] in Serum or Plasma
2093-3	−0.0000403	<1.0E-17	Cholesterol [Mass/volume] in Serum or Plasma
2500-7	−0.0000104	<1.0E-17	Iron binding capacity [Mass/volume] in Serum or Plasma
2498-4	−0.0000533	1.99E-10	Iron [Mass/volume] in Serum or Plasma
2075-0	−0.000161	4.53E-08	Chloride [Moles/volume] in Serum or Plasma

LOINC_ID is a standardized identifier for the laboratory. The adjusted coefficient indicates the change in the likelihood of stroke given a 1 % increase in lab values. The *p*-value indicates the likelihood that the given correlation is due solely to chance. A negative adjusted coefficient indicates that higher test values lower the risk of stroke

### Correlation

To test if some lab values were only effective correlates of stroke because of their correlation to other laboratory values, we measured the 741 pairwise correlation coefficients between the 38 different laboratory tests that were statistically significant correlates of stroke. These correlation measurements are shown in Additional file 1: Table S2 and in the Additional file 2. The table shows strong correlations between a few variables, most notably between various types of cholesterol. These correlations were calculated using the Pearson method, and again using R.

### Relative risk

We calculated the relative risk of each laboratory value to determine how these laboratory values change a patient’s risk of stroke. Several of the laboratory tests had non-linear relationships to stroke risk, preventing us from using a linear model of risk. Relative risk calculations require two distinct groups, instead of a continuous scale, so patients were grouped according to quartile. The relative risk computed in Table 2 compares patients in the lowest quartile (bottom 25 % of patients) to patients in the top quartile (top 25 %). The 95 % confidence intervals on the relative risk are also included.

**Table 2:** Relative risk

LOINC_ID	Relative_Risk	95 % confidence interval low	95 % confidence interval high
Urea nitrogen [Mass/volume] in Serum or Plasma	5.61	5.05	6.23
Creatinine [Mass/volume] in Serum or Plasma	4.48	4.17	4.82
Natriuretic peptide B [Mass/volume] in Serum or Plasma	4.18	3.25	5.37
Prothrombin time (PT) in Platelet poor plasma by Coagulation assay	3.59	2.75	4.69
Microalbumin/Creatinine [Mass Ratio] in Urine	3.3	2.97	3.68
Glucose [Mass/volume] in Serum or Plasma	3.21	2.84	3.63
Glucose [Mass/volume] in Serum or Plasma --8 h fasting	2.89	2.61	3.19
Hemoglobin A1c/Hemoglobin.total in Blood	2.62	2.41	2.84
CD3 + CD8+ (T8 suppressor cells) cells [#/volume] in Blood	2.6	1.3	5.2
Microalbumin [Mass/volume] in Urine	2.5	2.27	2.75
Q-T interval corrected	2.28	2.03	2.55
Neutrophils/100 leukocytes in Blood by Automated count	2.17	2.01	2.35
Monocytes [#/volume] in Blood by Automated count	2.06	1.81	2.35
Neutrophils [#/volume] in Blood by Automated count	1.98	1.8	2.17
Glucose mean value [Mass/volume] in Blood Estimated from glycated hemoglobin	1.94	1.8	2.09
Eosinophils/100 leukocytes in Blood by Automated count	1.85	1.71	2.01
Carcinoembryonic Ag [Mass/volume] in Serum or Plasma	1.84	1.22	2.8
QRS duration	1.81	1.62	2.02
CD3 cells [#/volume] in Blood	1.75	0.87	3.49
Potassium [Moles/volume] in Serum or Plasma	1.64	1.56	1.73
T wave axis.frontal plane Reference beat	1.62	1.47	1.78
Alkaline phosphatase [Enzymatic activity/volume] in Serum or Plasma	1.47	1.33	1.62
Hemoglobin F/Hemoglobin.total in Blood by HPLC	1.4	0.52	3.72
Iron binding capacity [Mass/volume] in Serum or Plasma	0.21	0.19	0.22
Cholesterol in LDL [Mass/volume] in Serum or Plasma by calculation	0.27	0.26	0.28
Cholesterol non HDL [Mass/volume] in Serum or Plasma	0.28	0.26	0.29
Cholesterol in LDL [Mass/volume] in Serum or Plasma	0.31	0.29	0.33
Cholesterol [Mass/volume] in Serum or Plasma	0.32	0.31	0.33
Albumin [Mass/volume] in Serum or Plasma	0.33	0.31	0.36
Lymphocytes/100 leukocytes in Blood by Automated count	0.38	0.34	0.44
Hematocrit [Volume Fraction] of Blood by Automated count	0.46	0.44	0.47
Iron [Mass/volume] in Serum or Plasma	0.56	0.51	0.62
Cholesterol in HDL [Mass/volume] in Serum or Plasma	0.65	0.63	0.68
Sodium [Moles/volume] in Serum or Plasma	0.72	0.69	0.75
Chloride [Moles/volume] in Serum or Plasma	0.72	0.69	0.75
Carbon dioxide	0.78	0.75	0.82
Glomerular filtration rate/1.73 sq M.predicted by Creatinine-based formula (MDRD)	^a^		
Eosinophils [#/volume] in Blood by Automated count	^a^		

^a^ indicates the value is incalculable due to lack of stroke events in 1st or 4th quartile

### Survival graphs

Kaplan-Meier survival graphs provide another measurement of the impact of laboratory values on the chance of stroke. The time until a stroke occurs is shown for each quartile as well as the 5^th^ and 95^th^ percentile of records. The laboratory test values that correspond to the indicated percentiles are given in Table 3. Note that most patients, regardless of lab values, did not suffer a stroke during this time period, and thus the survival plots remain above 90 %.

**Table 3:** The relationship between population percentile levels and absolute measurements of the 38 different laboratory tests

LOINC_ID	0.05	0.25	0.5	0.75	0.95	Units
Creatinine [Mass/volume] in Serum or Plasma	0.58	0.7	0.89	1.04	1.55	mg/dL
Albumin [Mass/volume] in Serum or Plasma	3	3.65	4	4.21	4.7	g/dL
Potassium [Moles/volume] in Serum or Plasma	3.5	3.8	4.1	4.3	4.8	mmol/L
Hemoglobin A1c/Hemoglobin.total in Blood	5.3	5.7	6.2	7.2	9.8	%
Neutrophils [#/volume] in Blood by Automated count	1.9	3	4.1	5.45	8.7	10^3/uL
Carbon dioxide	23	25	27	28	31	mmol/L
Hematocrit [Volume Fraction] of Blood by Automated count	32.4	37.2	40.2	43.1	47.25	%
Urea nitrogen [Mass/volume] in Serum or Plasma	8	11	15	20	39.5	mg/dL
Lymphocytes/100 leukocytes in Blood by Automated count	12.1	21.6	28.3	35	46.3	%
Cholesterol in HDL [Mass/volume] in Serum or Plasma	32	41	49	58	77	mg/dL
Neutrophils/100 leukocytes in Blood by Automated count	41.1	53.2	60.6	67.8	79	%
Sodium [Moles/volume] in Serum or Plasma	134	137	139	140	143	mmol/L
Cholesterol in LDL [Mass/volume] in Serum or Plasma by calculation	56	82	104	129	170	mg/dL
Cholesterol in LDL [Mass/volume] in Serum or Plasma	57	82	104	129	171	mg/dL
Cholesterol non HDL [Mass/volume] in Serum or Plasma	78	106	131	160	209	mg/dL
Glucose [Mass/volume] in Serum or Plasma --8 h fasting	77	87	95	104	143	mg/dL
Cholesterol [Mass/volume] in Serum or Plasma	123	155	181	210	258	mg/dL
Glucose [Mass/volume] in Serum or Plasma	73	85	94	107	179	mg/dL
Glucose mean value [Mass/volume] in Blood Estimated from glycated hemoglobin	107	120	138	167	232	mg/dL
Iron binding capacity [Mass/volume] in Serum or Plasma	246	311	354	400	471	ug/dL
Microalbumin [Mass/volume] in Urine	3.6	7.1	17.8	61.1	747.6	mg/dL
Microalbumin/Creatinine [Mass Ratio] in Urine	2.7	5.9	14.8	54.2	765.6	
Glomerular filtration rate/1.73 sq M.predicted by Creatinine-based formula (MDRD)	34	58	71	80	88	mL/min/1.73 m2
CD3 + CD8+ (T8 suppressor cells) cells [#/volume] in Blood	343	597	829	1128	1768	10^9/L
Prothrombin time (PT) in Platelet poor plasma by Coagulation assay	12.1	12.7	13.3	14.2	26	seconds
Hemoglobin F/Hemoglobin.total in Blood by HPLC	0.2	0.3	0.5	0.7	2	%
Monocytes [#/volume] in Blood by Automated count	0.3	0.4	0.5	0.6	1	10^3/uL
Eosinophils [#/volume] in Blood by Automated count	0	0	0.1	0.2	0.5	10^3/uL
Q-T interval corrected	387	410	427	444	476.5	ms
QRS duration	72	82	88	96	128	ms
Natriuretic peptide B [Mass/volume] in Serum or Plasma	13	34	129	337	1099	pg/mL
Eosinophils/100 leukocytes in Blood by Automated count	0.3	1.1	2	3.2	6.4	%
Iron [Mass/volume] in Serum or Plasma	23	52	77	105	158	ug/dL
Alkaline phosphatase [Enzymatic activity/volume] in Serum or Plasma	40	54	67	84	145	Units/L
CD3 cells [#/volume] in Blood	744	1201	1541	1940	2755	cells/uL
Chloride [Moles/volume] in Serum or Plasma	98	102	104	105	108	mmol/L
T wave axis.frontal plane Reference beat	−6	24	41	56	83	degree(angle)
Carcinoembryonic Ag [Mass/volume] in Serum or Plasma	1.1	1.6	2.5	4.8	59.7	ng/mL

**Fig. 2:**
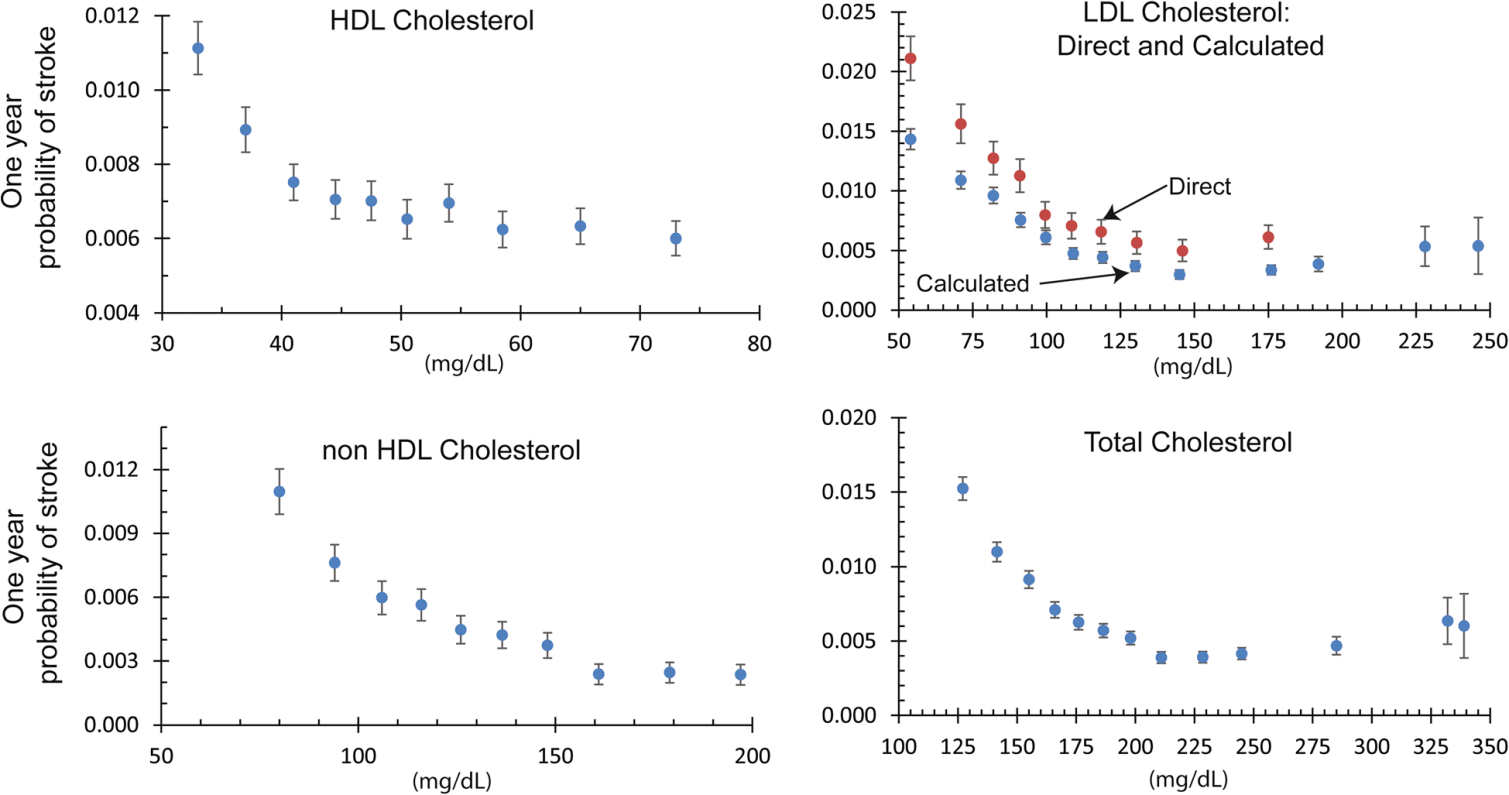
This figure presents the relationship between serum cholesterol levels and the risk of stroke measured from the electronic medical records of 2.4 million patients over a two year time span. Error bars indicate 95 % confidence intervals. The LDL levels calculated with the Friedewald equation are known to be systematically lower than direct measurements of LDL cholesterol [49]

Finally, we saw significant correlation in cholesterol levels: total cholesterol, HDL, non-HDL and LDL. Higher levels of all of these analytes were correlated with a lower risk of stroke. A detailed graph showing the relationship between cholesterol levels and the risk of stroke is shown in Fig. 2.

## Discussion

We identified 38 tests that effectively correlate with stroke. These 38 tests included three from electrocardiogram measurements, two from urine tests, and 33 from blood, serum and/or plasma. Many of the 33 blood tests are part of standard panels, including 12 that are typically included in (or calculated from) a comprehensive metabolic panel and 14 from a complete blood count with differential and 5 that are included in a lipid panel. The other two blood tests are used to diagnose heart failure (natriuretic peptide B, also known as BNP) and to monitor tumor progression (carcinoembryonic antigen, also known as CEA).

These 38 tests that proved to be correlated with stroke both reproduced previously known predictors of stroke and introduce several novel associations. The previously known correlates include kidney dysfunction and atrial fibrillation [25, 26].

We identified several measures of kidney function as significant correlate of stroke. Glomerular filtration rate is a standard measurement of renal function [27]. It is a calculated value based upon serum levels of creatinine, urea nitrogen, and albumin along with demographic factors: age, sex, and race [28]. Creatinine has been previously recognized as a stroke predictor [29, 30].

**Fig. 3:**
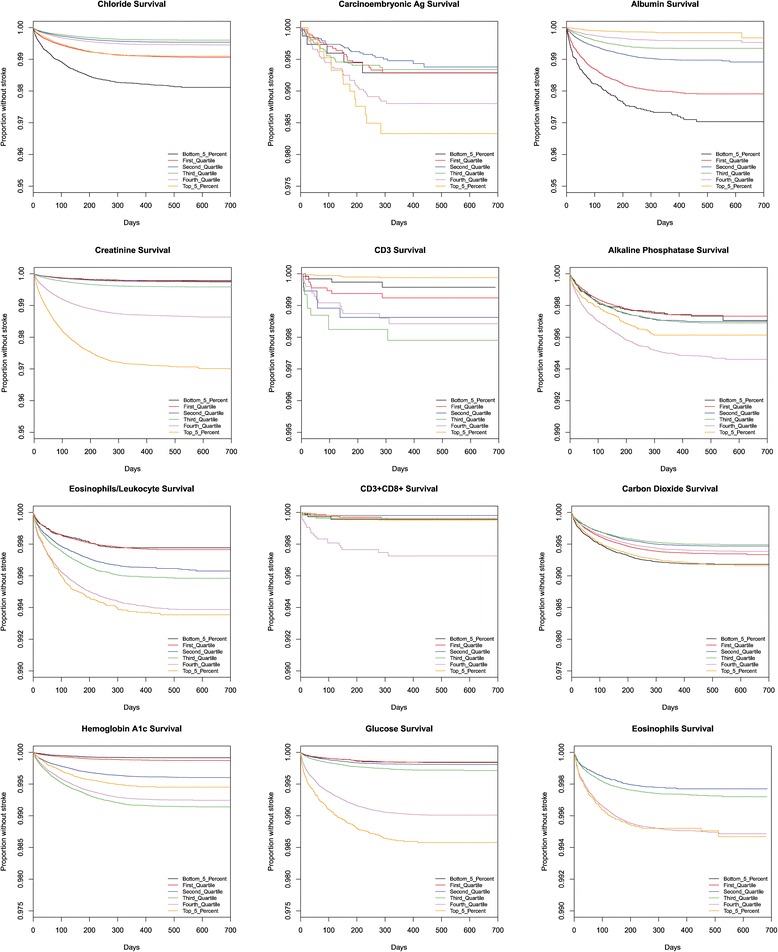
This figure presents Kaplan Meier survival graphs for 38 different laboratory tests. In each case, the graphs show the survival (percentage of the population who have not been diagnosed with a stroke) as a function of time. The population is broken up into five categories corresponding to those in the 5%, 25%, 50%, 75%, and 95%. The test values that correspond to those percentiles are given in Table 3. The 38 different survival graphs are separated into three panels: Fig 3, Fig 4, and Fig 5

**Fig. 4:**
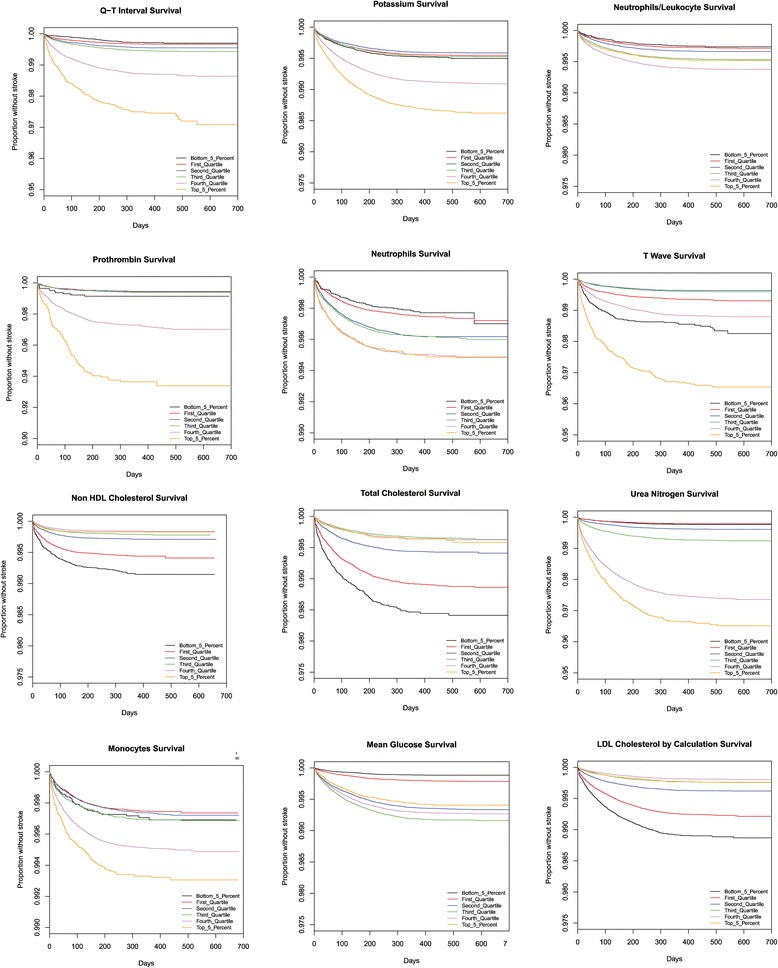
This figure presents Kaplan Meier survival graphs for 38 different laboratory tests. In each case, the graphs show the survival (percentage of the population who have not been diagnosed with a stroke) as a function of time. The population is broken up into five categories corresponding to those in the 5%, 25%, 50%, 75%, and 95%. The test values that correspond to those percentiles are given in Table 3. The 38 different survival graphs are separated into three panels: Fig 3, Fig 4, and Fig 5

**Fig. 5:**
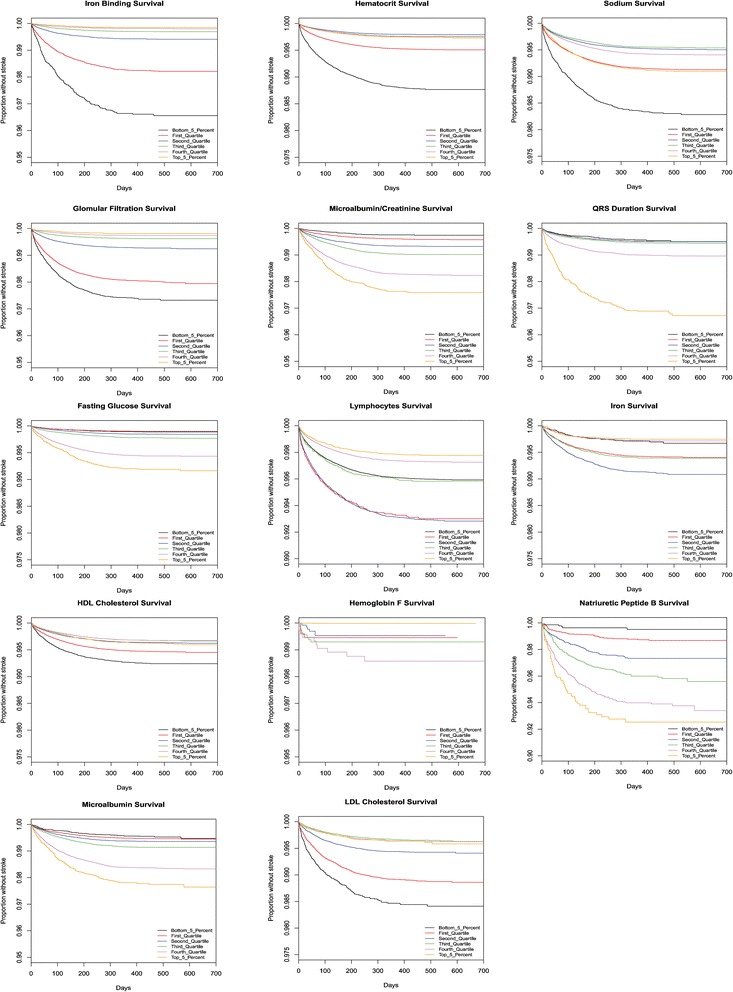
This figure presents Kaplan Meier survival graphs for 38 different laboratory tests. In each case, the graphs show the survival (percentage of the population who have not been diagnosed with a stroke) as a function of time. The population is broken up into five categories corresponding to those in the 5%, 25%, 50%, 75%, and 95%. The test values that correspond to those percentiles are given in Table 3. The 38 different survival graphs are separated into three panels: Fig 3, Fig 4, and Fig 5

The stroke prediction model [3] based upon the Cardiovascular Health Study [9] includes only one laboratory value, serum creatinine levels. The authors divided the population into two groups, split with serum creatinine levels above or below 1.25 mg/dL. In contrast, we found serum creatinine levels to be a strong correlate with continuous range from less than 0.58 mg/dL to greater than 1.74 mg/dL as shown in Figs. 3, 4 and 5.

The BNP test is used to diagnose congestive heart failure [31]. High levels of BNP (greater than 480 pg/ml) suggest congestive heart failure, while low levels (less than 100 pg/ml) rule it out [32]. BNP has been shown to predict mortality [33, 34]. We found BNP to be a particularly good correlate of stroke even at low and intermediate levels. We saw significant differences in the prognosis for stroke even between those who had BNP levels less than 13 pg/ml and those who tested less than 34 pg/ml.

Three parameters measured by electrocardiograms also appear in Table 1, including the Q-T interval, the QRS duration, and the T wave axis. Increases in both Q-T interval and QRS duration were positively correlated with stroke incidence. T wave axis was also more positively correlated as the axis shifted rightward (i.e., increased).

Of the 38 tests that were correlated with stroke, many fell into distinct groups of similar function. Four laboratory tests associated with diabetes all correlated positively with stroke, which supports previous research on the subject [35]. Similarly, laboratory results that might reflect impaired kidney function, such as creatinine, urea nitrogen, and potassium, are also positively correlated with stroke chance. This also agrees with previous work showing that patients with Chronic Kidney Disease are associated with a higher risk of stroke [36].

Interestingly, higher levels for non-HDL cholesterol are significant risk factors for cardiac disease [37], but not necessarily for stroke. The MRFIT study of 350,977 men over six years found that lower levels of total cholesterol lead to a higher risk of death by intracranial hemorrhage, while higher levels did lead to an increased risk of non-hemorrhagic stroke [38]. Men with serum cholesterol levels less than 160 mg/dL had three times the risk of intracranial hemorrhage compared to those with higher cholesterol levels. The more recent Physicians’ Health Study, 22,071 men followed for 11 years, found no significant correlation between total cholesterol, HDL cholesterol, or triglycerides and ischemic stroke [39].

Our findings showed that higher levels of total cholesterol, HDL, LDL, and non-HDL cholesterol were correlated with significantly decreased incidence of stroke. Although other research has shown that high HDL is correlated with decreased risk of stroke, the research on total cholesterol and LDL has been more mixed. Our results for these tests are highly significant, and this finding is interesting because many of the patients are likely treated with statins, which are known to reduce stroke risk while lowering LDL and total cholesterol [40]. It is possible that stroke risk depends on long-term cholesterol levels, and the use of statins suddenly reduces cholesterol levels, thus distorting this data. However, since we did not include medications in our assessment, we are unable to determine what relationship treatment might have on interpreting these findings.

The significance of the EKG correlations in our results in unclear because the clinical assessments of the EKGs were not available in the discrete data analyzed. It is possible that increases in the Q-T interval and QRS duration being correlated with increase in stroke was due to underlying cardiac disease and secondary cardiac manifestations of that disease like bundle branch blocks or the use of antiarrhythmic medication. The positive correlation of rightward axis deviation is possibly due to similar causes, or possibly associated with COPD and right-sided heart failure or right ventricular hypertrophy. Further assessment of this relationship would help clarify the significance of this correlation.

The results for iron-related labs are inconsistent. High TIBC is negatively correlated with stroke, but high iron is also negatively correlated, although at a much lower significance level. Since high TIBC is a better correlate, it could imply that iron deficiency is correlated with increased risk of stroke, although we would also expect low iron in that situation. The literature on the association of iron with stroke appears to be mixed, with a recent study [41] on a specific population of patients showing positive correlation, and other studies showing negative [42] or mixed [43] correlations. Further analysis of iron’s association with stroke may be helpful.

Albumin was the strongest negative correlate for stroke in our results. This observation is consistent with numerous studies that showed a beneficial effect of high albumin on risk of stroke [44–46].

We identified common laboratory blood tests that correlate with stroke. Some were positively associated (neutrophil count and percent, CD3 + CD8+ T8 suppressor cells, monocytes, eosinophils, and CD3 cells), while others were negatively correlated (hematocrit, lymphocytes). Given the numerous clinical reasons for these blood tests to be both high and low across inpatient and outpatient encounters, it is difficult to determine any likely justification for the correlation from these readings.

This analysis has a number of limitations. The discrete data used is only from a one-year time period, and may benefit from expanding to additional years. We selected for review only patients with KP health insurance that was continuous, thereby limiting the assessment to only insured patients. This selection likely underrepresents for people that were uninsured or partially insured for any reason. We did not include claims data to identify KP members that were diagnosed with strokes outside of the KP network of care or outside of this time period, and therefore may not include all KP members that had strokes during that timeframe or before the beginning of the timeframe. Our method of taking the median laboratory value when multiple results exist during a month may artificially normalize abnormal readings, doesn’t fully take into account the potential impact of acute care events, and even then isn’t necessarily independent. Other methods exist for considering time correlations that could be used in a similar assessment [47].

We did not include medication data in our analysis, which could have a significant impact on some of our findings. In particular, the significance of the cholesterol tests on stroke is likely complicated by the high rate of LDL control using cholesterol lowering medications, like statins, at KP, and their ability to reduce the risk of strokes [40, 48]. Additional analysis that factored in medication use would likely raise additional findings for consideration.

We included both hemorrhagic and non-hemorrhagic strokes in our analysis, and did not differentiate between the two. This allowed us to compare our results to other landmark studies [1, 2]. Since the risk factors for these two conditions are different, and the number of non-hemorrhagic strokes is significantly higher than hemorrhagic strokes, our analysis may not accurately reflect the correlation between these lab tests and hemorrhagic strokes. Further assessment of each sub-group would be an important area for further analysis. Over 95 % of the strokes analyzed were non-hemorrhagic.

We used ICD-9 diagnoses documented in the medical record to identify patients with a stroke. Since this included problem list diagnoses, it is possible that patients were included that had a history of stroke and not a current stroke event if that diagnosis was added to the problem list during our timeframe. Also, a diagnosis of stroke is only appropriate as an acute diagnosis, but we did not limit our selection to only acute encounters, and therefore could have over-selected patients into our cohort who had previously had strokes. We could have limited our selection of patients to only first-time stroke patients using more historic data, and not including patients with diagnoses representing side effects of strokes, but did not do so.

## Conclusion

In conclusion, we identified several dozen independent laboratory tests that are strong correlates of stroke. These laboratory tests could be combined to provide a short-term (one year) measure of the probability that a patient will be diagnosed with a stroke.

## Abbreviations

BNP, B-type natriuretic peptide; CD3, cluster of differentiation; CEA, carcinoembryonic antigen; COPD, chronic obstructive pulmonary disease; EKG, electrocardiogram; HDL, high density lipoproteins; ICD, International Classification of Disease; KP, Kaiser Permanente; LDL, low density lipoproteins; LOINC, logical observation identifiers names and codes; MRFIT, multiple risk factor intervention trial; PHI, personal health identifiers; TIBC, total iron binding capacity

## Additional files

Additional file 1: Table S1. Racial/ethnic distribution of patients in the dataset. **Table S2.** Correlations between different laboratory tests. (PDF 277 kb) Additional file 2: The Relative Risk (with confidence intervals), the correlations computed between different tests, the description and range of observed values for each test. (XLSX 48 kb)
