# Supplementary material for: Laboratory tests as short-term correlates of stroke
Source: BMC Neurol. 2016 Jul 21;16:112. doi: 10.1186/s12883-016-0619-y (PMC4955202; doi:10.1186/s12883-016-0619-y)
Supplement: Additional file 1: Table S1. — Racial/ethnic distribution of patients in the dataset. Table S2. Correlations between different laboratory tests. (PDF 277 kb) [file 12883_2016_619_MOESM1_ESM.pdf]

CATEGORY	NUMBER	PERCENTAGE
**HISPANIC/LATINO**	682,670	28.30%
**WHITE**	598,491	24.81%
**NO INFORMATION**	334,681	13.87%
**WHITE - OTHER WHITE OR EUROPEAN**	182,863	7.58%
**BLACK/AFRICAN AMERICAN**	176,913	7.33%
**ASIAN**	172,327	7.14%
**DECLINE TO STATE**	88,249	3.66%
**NOT RECORDED**	76,589	3.18%
**WHITE - WESTERN EUROPE**	29,112	1.21%
**BLACK - OTHER BLACK**	29,100	1.21%
**UNKNOWN - PATIENT REFUSAL**	24,469	1.01%
**NATIVE HAWAIIAN/OTHER PACIFIC ISLANDER**	16,639	0.69%

Supplemental Table I. The racial/ethnic distribution of patients in the dataset.

Supplemental Table II

LOC-ID	1751-7	4544-3	2028-9	2951-2	736-9	2085-9	13457-7	43396-1	2089-1	2093-3
1751-7	1.00	0.41	0.00	0.17	0.21	0.10	0.12	0.11	0.15	0.15
4544-3		1.00	0.13	0.14	0.00	-0.15	0.14	0.17	0.19	0.13
2028-9			1.00	0.18	0.06	0.06	0.02	-0.01	0.01	0.01
2951-2				1.00	0.12	0.02	0.04	-0.01	0.04	0.00
736-9					1.00	0.05	0.11	0.10	0.11	0.10
2085-9						1.00	0.14	-0.03	0.09	0.30
13457-7							1.00	0.87	0.62	0.91
43396-1								1.00	0.69	0.91
2089-1									1.00	0.76
2093-3										1.00
2500-7										
33914-3										
14959-1										
14957-5										
2345-7										
27353-2										
17865-7										
770-8										
3094-0										
751-8										
4548-4										
2160-0										
2823-3										
14135-8										
5902-2										
42246-9										
742-7										
711-2										
8636-3										
8633-0										
30934-4										
713-8										
2498-4										
6768-6										
8122-4										
2075-0										
18518-1										
2039-6										

2500-7	33914-3	14959-1	14957-5	2345-7	27353-2	17865-7	770-8	3094-0	751-8	4548-4	2160-0	2823-3	14135-8
0.31	0.20	-0.32	-0.33	-0.17	-0.14	-0.10	-0.16	-0.18	-0.14	-0.15	-0.16	0.01	0.02
-0.03	0.28	-0.17	-0.14	-0.02	-0.01	0.04	0.00	-0.22	0.01	0.01	-0.04	0.08	0.06
-0.03	0.15	-0.11	-0.10	-0.05	-0.03	0.01	-0.07	-0.14	-0.12	-0.03	-0.03	-0.05	-0.03
-0.04	0.02	-0.04	-0.03	-0.23	-0.24	-0.18	-0.11	0.03	-0.12	-0.26	0.00	0.07	-0.05
0.15	0.18	-0.10	-0.10	-0.14	-0.04	-0.06	-0.95	-0.17	-0.64	-0.04	-0.12	-0.02	0.46
0.13	0.06	-0.02	-0.04	-0.14	-0.20	-0.16	-0.03	-0.10	-0.12	-0.19	-0.13	-0.05	-0.17
0.08	0.15	0.03	0.04	-0.12	-0.17	-0.09	-0.08	-0.15	-0.08	-0.14	-0.08	-0.05	-0.01
0.09	0.10	0.08	0.09	-0.02	-0.02	-0.02	-0.07	-0.11	-0.03	-0.01	-0.06	-0.05	0.06
0.16	0.18	0.02	0.02	-0.06	-0.08	-0.09	-0.07	-0.19	-0.02	-0.07	-0.12	-0.08	0.02
0.13	0.13	0.07	0.07	-0.04	-0.10	-0.05	-0.07	-0.14	-0.06	-0.07	-0.10	-0.06	-0.01
1.00	0.36	-0.23	-0.23	-0.13	-0.04	-0.07	-0.10	-0.37	-0.05	-0.05	-0.30	-0.15	-0.05
	1.00	-0.29	-0.27	-0.15	-0.08	-0.10	-0.14	-0.70	-0.11	-0.09	-0.61	-0.19	0.01
		1.00	0.83	0.05	0.06	0.04	0.08	0.32	0.06	0.07	0.37	0.09	-0.02
			1.00	0.05	0.08	0.06	0.09	0.30	0.07	0.09	0.35	0.08	-0.02
				1.00	0.67	0.57	0.14	0.15	0.14	0.72	0.09	0.08	0.04
					1.00	0.74	0.04	0.12	0.12	0.99	0.02	0.10	0.05
						1.00	0.06	0.11	0.09	0.78	0.06	0.07	0.04
							1.00	0.11	0.07	0.03	0.07	-0.02	-0.42
								1.00	0.06	0.13	0.63	0.31	0.02
									1.00	0.11	0.04	-0.03	0.00
										1.00	0.03	0.10	0.03
											1.00	0.18	0.00
												1.00	0.03
													1.00

5902-2	42246-9	742-7	711-2	8636-3	8633-0	30934-4	713-8	2498-4	6768-6	8122-4	2075-0	18518-1	2039-6
-0.26	0.01	-0.12	-0.07	-0.17	-0.04	-0.15	-0.06	0.24	-0.20	0.14	-0.01	-0.02	-0.04
-0.14	-0.21	0.03	0.02	-0.16	0.07	-0.19	0.02	0.37	-0.10	0.01	-0.05	-0.04	-0.08
0.04	-0.04	-0.04	0.00	-0.04	0.06	0.01	0.05	0.07	-0.09	-0.05	-0.34	0.01	-0.02
-0.04	0.02	-0.11	0.01	-0.03	0.00	-0.02	0.05	0.06	-0.06	-0.02	0.56	-0.01	-0.04
-0.19	0.17	-0.20	0.02	-0.13	-0.11	-0.18	0.12	0.13	0.00	0.47	0.14	-0.02	-0.05
-0.08	-0.03	-0.13	-0.10	-0.01	-0.14	-0.07	-0.05	0.09	-0.06	-0.12	-0.04	0.06	0.00
-0.15	-0.02	-0.09	-0.04	-0.07	-0.12	-0.16	-0.02	0.07	-0.03	0.01	0.02	-0.02	0.00
-0.15	-0.05	-0.06	-0.01	-0.05	-0.11	-0.17	-0.02	0.08	0.00	0.07	-0.03	-0.03	0.04
-0.16	-0.07	-0.06	-0.03	-0.07	-0.14	-0.14	-0.04	0.07	-0.02	0.05	0.01	-0.02	0.02
-0.17	-0.06	-0.09	-0.04	-0.04	-0.15	-0.20	-0.03	0.10	-0.03	0.02	-0.05	-0.01	0.01
-0.19	-0.08	-0.07	-0.06	-0.06	-0.13	-0.10	-0.08	-0.05	-0.06	0.08	-0.01	-0.04	-0.06
-0.13	0.01	-0.11	-0.10	-0.16	-0.12	-0.29	-0.08	0.12	-0.09	0.04	-0.01	-0.06	0.01
-0.01	-0.03	0.01	0.05	0.06	0.03	0.12	0.05	-0.06	0.05	-0.05	0.05	0.04	0.01
-0.01	-0.02	0.02	0.05	0.07	0.04	0.11	0.04	-0.05	0.04	-0.05	0.06	0.04	0.01
0.04	0.02	0.04	0.02	0.13	0.06	0.03	-0.01	-0.05	0.09	0.01	-0.24	0.01	0.08
0.00	-0.12	0.08	0.09	0.12	0.04	0.00	0.04	-0.14	0.11	0.06	-0.25	0.01	0.04
0.03	-0.02	0.06	0.06	0.11	0.07	0.03	0.02	-0.03	0.09	0.02	-0.20	0.00	0.03
0.14	-0.17	0.08	-0.19	0.11	0.06	0.14	-0.32	-0.13	-0.01	-0.42	-0.12	0.01	0.05
0.11	-0.05	0.09	0.11	0.14	0.17	0.31	0.11	-0.08	0.04	-0.01	0.03	0.06	-0.02
0.08	-0.05	0.46	0.02	0.11	0.01	0.02	-0.23	-0.17	0.05	0.08	-0.15	0.00	0.05
0.01	-0.09	0.08	0.08	0.13	0.04	0.02	0.04	-0.13	0.12	0.04	-0.27	0.01	0.03
0.05	-0.06	0.07	0.09	0.08	0.12	0.28	0.09	-0.03	0.02	-0.04	-0.05	0.03	0.00
0.08	0.02	0.04	0.07	-0.08	0.08	0.10	0.09	0.02	0.02	0.02	0.09	0.04	-0.01
-0.06	0.24	0.26	0.09	0.01	-0.04	-0.05	-0.02	0.04	0.01	0.80	-0.01	-0.08	0.38
1.00	0.17	0.12	0.01	0.19	0.19	0.17	0.02	-0.10	0.08	-0.12	-0.04	-0.03	-0.02
	1.00	0.11	0.06	0.06	0.00	-0.14	0.03	0.10	0.20	0.13	0.03	-0.03	-0.09
		1.00	0.14	0.06	0.07	0.03	-0.04	-0.09	0.06	0.32	-0.14	0.01	0.05
			1.00	0.03	0.06	-0.01	0.87	-0.04	0.05	0.13	0.00	0.02	-0.03
				1.00	0.32	0.30	0.00	-0.10	0.08	0.02	-0.08	0.04	0.01
					1.00	0.24	0.06	0.00	-0.01	-0.06	-0.03	0.02	-0.03
						1.00	0.00	-0.08	0.11	-0.01	-0.07	0.04	-0.05
							1.00	0.01	0.03	-0.04	0.05	0.02	-0.02
								1.00	-0.05	0.06	0.00	0.00	-0.06
									1.00	0.00	-0.05	0.01	0.18
										1.00	-0.01	-0.12	0.07
											1.00	-0.02	-0.04
												1.00	0.01
													1
